# Descriptive characteristics of hospitalized adult smokers and never-smokers with COVID-19

**DOI:** 10.18332/tid/122759

**Published:** 2020-05-28

**Authors:** Mohammad Ebrahimi Kalan, Hassan Ghobadi, Ziyad Ben Taleb, Kenneth D. Ward, Davoud Adham, Somaieh Matin, Mehdi Fazlzadeh, Sajjad Narimani

**Affiliations:** 1 Department of Epidemiology, Robert Stempel College of Public Health and Social Work, Florida International University, Miami, United States; 2 Department of Internal Medicine, Pulmonary Division, School of Medicine, Ardabil University of Medical Sciences, Ardabil, Iran; 3 Department of Kinesiology, College of Nursing and Health Innovation, University of Texas at Arlington, Arlington, United States; 4 Division of Social and Behavioral Sciences, School of Public Health, The University of Memphis, Memphis, United States; 5 Public Health Department, School of Health, Ardabil University of Medical Sciences, Ardabil, Iran; 6 Environmental Health Department, School of Health, Ardabil University of Medical Sciences, Ardabil, Iran; 7 Department of Environmental Health Engineering, School of Public Health, Tehran University of Medical Sciences, Tehran, Iran; 8 Nursing and Midwifery, Social Determinants of Health Research Center, Ardabil University of Medical Sciences, Ardabil, Iran

**Keywords:** waterpipe smoking, cigarettes smoking, COVID-19

**Dear Editor,**

A meta-analysis done by Guo^1^ that was published in *Tobacco Induced Diseases*, reported a pooled odds ratio of 2.20, concluding that active smoking is significantly associated with the risk of severe COVID-19. Another current meta-analysis reported greater odds of COVID-19 progression among smokers compared to never smokers^2^. Most of the studies in these meta-analyses were from China and focused only on cigarette smoking^1,2^. Here, we describe characteristics of tobacco use among 193 confirmed COVID-19 patients who were interviewed during their hospitalization from 15 March to 15 April 2020, in the Imam-Khomeini Hospital of Ardabil University of Medical Sciences (ArUMS) in Iran. The protocol was approved by the Institutional Review Board of Ardabil University of Medical Sciences (Approval ID: IR.ARUMS.REC.1399.044) and verbal informed consent was obtained from the patients.

All patients tested positive for SARS-CoV-2 by nasopharyngeal swabs using real-time reverse-transcription-polymerase-chain-reaction (rRT-PCR) assay; they were at least 18 years old and willing to participate in an approximately 5-min interview. Interviews were administrated in the first two days of hospitalization to collect information regarding demographics, COVID-19-associated symptoms, and use of tobacco products including waterpipe (WP, hookah), cigarettes, and e-cigarettes.

As shown in Table 1, 15 (7.8%) and 14 (7.3%) of the patients reported current (past-month) WP or cigarette use, respectively. No patients were dual WP/cigarette or e-cigarette users. Of the 14 cigarette smokers, 2 had cardiovascular disease (CVD), 1 chronic respiratory illness, 1 diabetes, 2 other conditions (e.g. kidney illness or rheumatoid arthritis), and 4 reported having >1 chronic condition. Among 15 WP smokers, 3 reported having CVD, 1 other condition, and 2 having >1 chronic condition. The average time between the onset of symptoms and hospitalization was approximately 4 days for WP smokers, 3 days for cigarette smokers, and 5 days for never-smokers. More than half (n=8) of cigarette smokers and 40% (n=6) of WP smokers reported their COVID-19 symptoms as severe compared to 22% (n=36) of never-smokers.

**Table 1:** Descriptive characteristics of adult smokers and never-smokers hospitalized with COVID-19 from 15 March to 15 April 2020 at Imam-Khomeini Hospital of Ardabil University of Medical Sciences, Ardabil, Iran (N=193 )

*Characteristics*	*Never-smokers ^a^ (N=164)*	*Waterpipesmokers ^b^ (N=15)*	*Cigarette smokers ^c^ (N=14)*	*p*	*Total (N=193)*
	*n (%) /mean±SD*	*n (%) / mean±SD*	*n (%) /mean±SD*		*n (%) /mean±SD*
**Age** (years)	52.2±14.8	52.5±11.8	58.2±16.5	0.344	52.6±14.8
**Gender**					
Male	95 (57.9)	15 (100.0)	13 (92.9)	0.001	123 (63.7)
Female	69 (42.1)	0 (0.00)	1 (7.1)		70 (36.3)
**Education**					
Illiterate	25 (15.2)	2 (13.3)	3 (21.4)	0.697	30 (15.5)
≤High school	111 (67.7)	11 (73.3)	7 (50.0)		129 (66.8)
Academic	28 (17.1)	2 (13.3)	4 (28.6)		34 (17.6)
**History of chronic diseases**					
None	91 (55.5)	9 (60.0)	4 (28.6)	0.482	104 (53.9)
CVD	24 (14.6)	3 (20.0)	2 (14.3)		29 (15.0)
Pulmonary	7 (4.3)	0 (0.0)	1 (7.1)		8 (4.1)
Diabetes	11 (6.7)	0 (0.0)	1 (7.1)		12 (6.2)
Other	13 (7.9)	1 (6.7)	2 (14.3)		16 (8.3)
>1 condition	18 (11.0)	2 (13.3)	4 (28.6)		24 (12.4)
Days between symptoms onset and	5.0±2.0	4.0±1.3	3.1±1.0		4.8±1.9
hospitalization					
**Symptoms** (yes vs no)					
Dry cough	138 (84.1)	11 (73.3)	13 (92.9)	0.353	162 (83.9)
Fever	108 (65.9)	10 (66.7)	11 (78.6)	0.625	129 (66.8)
Body pain	81 (49.4)	12 (80.0)	5 (35.7)	0.038	98 (50.8)
Shortness of breath	90 (54.9)	3 (20.0)	8 (57.1)	0.033	101 (52.3)
**How do you define your symptoms**					
Mild	51 (31.3)	4 (26.7)	1 (7.1)	0.030	56 (29.2)
Moderate	76 (46.6)	5 (33.3)	5 (35.7)		88 (44.8)
Severe	36 (22.1)	6 (40.0)	8 (57.1)		50 (26.0)
**Cigarette smoking frequency/month**					
<10	–	–	3 (21.4)	–	3 (21.4)
10–20	–	–	7 (50.0)		7 (50.0)
>20	–	–	4 (28.6)		4 (28.6)
**WP smoking pattern**					
Daily/weekly	–	8 (53.3)	–	–	8 (53.3)
Monthly	–	7 (46.7)	–		7 (46.7)
**Outcome of disease^d^**					
Discharged in <5 days	102 (62.2)	11 (73.3)	9 (64.3)	0.002	122 (63.2)
Transferred to ICU	60 (36.6)	1 (6.7)	5 (35.7)		66 (34.2)
Died	2 (1.2)	3 (20.0)	0 (0.0)		5 (2.6)

CVD: cardiovascular diseases. ICU: intensive care unit.

a Never-smokers were defined as individuals who have never used any kind of tobacco product.

b Waterpipe smokers were those who smoked WP at least once in past 30 days preceding this study.

c Cigarette smokers were those who smoked cigarette at least once in past 30 days preceding this study.

d Approximately 10 days after the interview, outcome of disease was recorded from hospital records. According to hospital protocol, patients stayed for >5 days only if ICU care was needed, to keep beds free for new patients.

In line with a previous report^3^, the most common COVID-19 symptoms were fever and dry cough, which did not differ significantly between smokers (cigarettes or WP) and never-smokers. However, shortness of breath was reported more frequently by cigarette smokers compared to never-smokers and WP smokers. Body pain was reported more frequently among WP smokers compared to cigarette smokers and never-smokers.

Three out of the five deaths occurred among WP smokers with the remaining two deaths among never-smokers. When interviewed at the beginning of the study, all three WP smokers and 2 never-smokers who later died due to COVID-19 infection reported having chronic conditions.

It is suggested that WP smoking be regulated to restrict COVID-19 transmission^4,5^ and several countries accordingly have temporarily shut down WP cafes^4,6^. Berlin et al.^7^ highlighted that ‘lockdown may be an opportune moment to quit to reduce not only the smoker’s health risk but also that of his/her family members’. This recommendation might not be practical, however, in countries like Iran (the epicenter of COVID-19 in the Middle East), where WP smoking is culturally rooted, highly prevalent, and frequently occurs at home^8,9^. As social distancing restrictions are relaxed, it is critical to provide guidelines (e.g. best cleaning practices, hygiene standards) and increase awareness regarding the risks of sharing WP. Future studies investigating the association between smoking and COVID-19 should consider WP smoking as a potential conduit of infection for this new disease. These studies could answer critical questions about whether smoking WP in a café environment will increase the risk of contracting coronavirus, how café owners must sanitize WP components to reduce the risk of virus transmission, and whether sharing the same WP hose in a home environment between family members or friends increases the risk of suffering from serious symptoms due to COVID-19 illness.

The limitations of this study include the self-report of underlying medical conditions, tobacco smoking, and the severity of COVID-19-associated symptoms. Despite these limitations, this descriptive study is among the first to describe the patterns of WP use among COVID-19 patients. Our findings highlight the need for larger scale studies to further investigate the influence of smoking behavior on the severity and the prognosis of COVID-19.
